# Associations of community, famliy and early individual factors with body mass index z-scores trajectories among Chinese children and adolescents

**DOI:** 10.1038/s41598-021-93949-4

**Published:** 2021-07-15

**Authors:** Jing Liang, Si Zheng, Xuyang Li, Dianmin Xiao, Peigang Wang

**Affiliations:** 1 School of Health Sciences, Wuhan University, Wuhan, 430071 China; 2 Wuhan University Center for Population and Health Research, Wuhan, 430071 China; 3 Gannan Medical University, Ganzhou, 341000 China

**Keywords:** Risk factors, Weight management

## Abstract

The prevalence of childhood overweight and obesity is increasing. This study aimed to examine trajectories of BMI z-scores among Chinese children and the potential determinants including early individual, family and community factors. Group-based trajectory modeling was employed to identify BMI z-scores trajectories of children aged 2–18 years using the five waves data (2010, 2012, 2014, 2016, and 2018) of the China Family Panel Studies (CFPS). Multivariate logistic regression was conducted to determine the association between early individual, family, community factors and BMI z-scores trajectories of children. We identified three trajectories for boys and girls, named Class 1 as “not-overweight”, Class 2 as “persistent rapid descending but overweight during pre-school age”, and Class 3 as “rapid rising up to school age and then become-overweight” class. Macrosomia (OR 1.772; 95% CI 1.188–2.644) and being a single child (OR 2.038; 95% CI 1.453–2.859) were more likely to belong in Class 3 among boys. Girls living in the advantaged communities (OR 1.539; 95% CI 1.052–2.252), rural-living (OR 1.558; 95% CI 1.133–2.142) and with none social integration (OR 1.496; 95% CI 1.07–2.091) were more likely to belong in Class 2. There are heterogeneous BMI z-scores trajectories of children aged 2–18, and pre-school age is a critical window that could predict the long-term growth patterns. BMI z-scores trends need to be monitored during pre-school age, focusing on those at higher risk of later overweight obesity status, and targeted interventions at the early individual, family, community levels are essential.

## Introduction

The prevalence of childhood overweight and obesity is increasing, especially in developing countries^1^. According to the latest Chinese survey report, the prevalence of overweight and obesity was 19% among children aged 6–17 and 10.4% among children under 6^2^. It is estimated that by 2030, the direct economic cost of obesity-related chronic diseases will increase to 49.05 billion RMB per year^3^. Therefore, the trends in childhood overweight and obesity should be closely monitored. Estimating the patterns of different weight trajectories based on standardized BMI of children in the recent birth cohort, and taking effective measures can promote sustainable development of the society and economy.

The BMI z-score is often used to measure children’s overweight/obesity and to model relative children’s weight trajectories longitudinally^4^. A recent longitudinal study found that the average BMI z-scores trajectory decreased with age among Chinese children^5^. Another study used age- and sex-specific percentiles for BMI to define overweight/obesity, and found three different types of overweight/obesity trajectories among Chinese adolescents from 2010 to 2016^6^. A small number of studies on the trajectory of overweight/obesity in Chinese children are available. Nevertheless, some of them only studied the trajectory of average BMI z-scores^7^, the heterogeneity of trajectory was not considered. Although some other studies^6,8^ used dichotomous variables such as overweight or obesity to estimate different trajectories, they did not comprehensively demonstrate the process of weight change by using the continuous variable of BMI z-scores. In order to fill these gaps, it is necessary to use BMI z-scores to explore different types of weight trajectories of Chinese children, so as to put forward more targeted measures for different weight trajectories.

Numerous scholars have found many factors that affect children’s overweight/obesity. At the individual level, the development of obesity may start early in an individual’s life. It is affected by prenatal, perinatal, and postnatal environmental factors^9–11^. Previous research has established that preterm infants are at a higher risk of developing childhood obesity than term infants, as low birth weight is often accompanied by rapid postnatal weight gain^12^. Moreover, there is growing evidence that breastfeeding protects against subsequent obesity^13,14^. At the family level, a large number of studies indicated that childhood obesity has a significant association with specific family characteristics, the prevalence of childhood obesity is highest in single-parent families, in lower-income households, and mothers with less educated^15–17^. At the community level, neighbor’s income inequality and ethnic composition are related to childhood obesity^18,19^. It has been found that food, physical activity and built environment have a deep impact on children’s weight status. The higher density of convenience stores is associated with a higher BMI z-score^20^. Moreover, children residing in the community with higher walkability, better access to public transit, and parks are less likely to be overweight^21,22^. In addition, the higher social integration between neighbors, the lower prevalence of obesity^23^.

The social-ecological theory divides these factors into different levels: individual level, family level, community/school level, and suggests that prevention strategies should be formulated according to different levels^24,25^. However, there are no comprehensive researches on socio-ecological determinants of overweight/obesity in Chinses recently born children, especially focusing on the BMI z-scores trajectory. Therefore, we draw on data from a new longitudinal survey in China to examine the trajectories of BMI z-scores among children and the potential determinants based on the social-ecological theory, including early individual, family and community factors. We hope to propose a more comprehensive and targeted intervention strategy about children’s overweight and obesity.

## Results

### BMI z-scores trajectories

**Figure 1:**
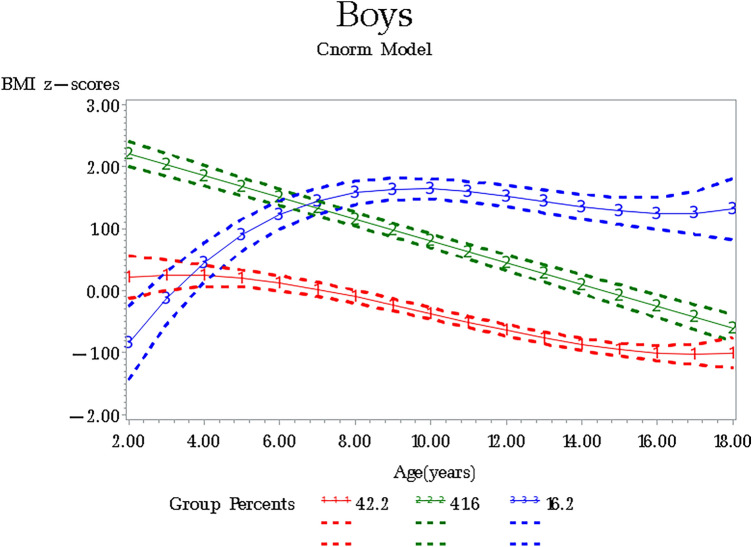
BMI z-scores trajectory groups among boys aged from 2 to 18, CFPS.

**Figure 2:**
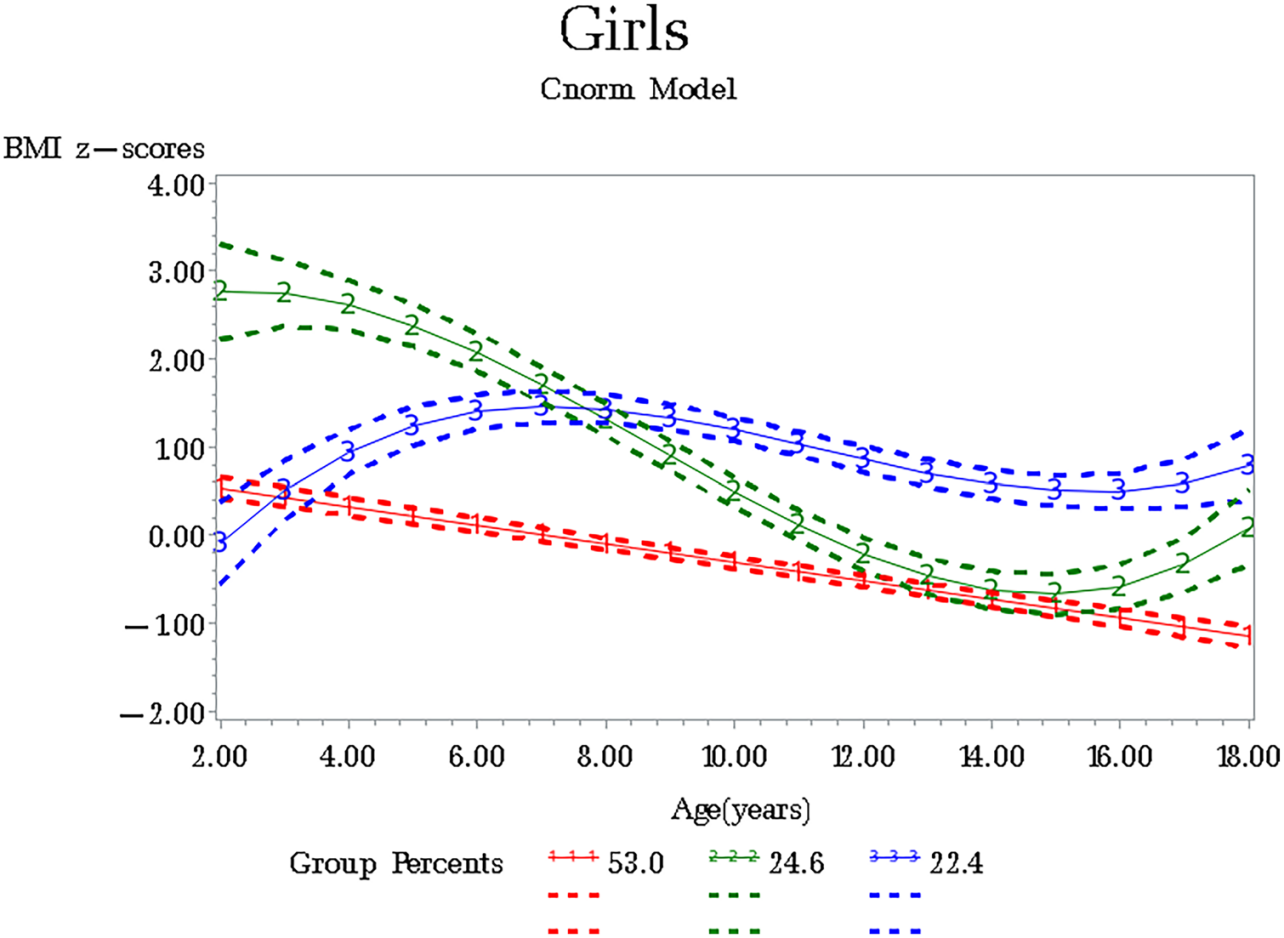
BMI z-scores trajectory groups among girls aged from 2 to 18, CFPS.

The number of classes and the shape of BMI z-scores trajectories were estimated from 1913 boys and 1607 girls, respectively. From a 1-class model to a 6-class model, the 5-class model for boys and the 4-class model for girls yielded an even lower BIC. However, considering the average posterior probabilities for each subgroup > 0.7, the 3-class model was selected as the best fitting model for boys and girls, respectively. Table 1 shows the fit statistics for the trajectory classes estimated for boys and girls in CFPS. Figures 1 and 2, respectively show BMI z-scores trajectories for the 3-class model and the sizes of the three classes for boys and girls. We have identified three trajectories in both boys and girls that were similar in their growth  patterns, but differed in their proportions. According to the relative position of the estimated three trajectories, we named Class 1 as “Not-overweight” (42.24% for boys, 52.98% for girls), Class 2 as “Persistent rapid descending but overweight during pre-school age” (41.55% for boys, 24.64% for girls), and Class 3 as “Rapid rising up to school age and then become-overweight ” (16.20% for boys, 22.38% for girls). Note that Class 3 had a stable pattern with overweight during puberty, especially among boys. Also, Tables 2 and 3 display the trajectory descriptions, the median posterior probabilities, and the growth parameter among boys and girls, respectively.

**Table 1:** Fit statistics for the trajectory classes estimated in the CFPS.

Class	Orders^a^	BIC^b^	2ΔBIC^c^	The average posterior probabilities^d^
**Boys**				
1	2	− 13,257.92		1
2	3/1	− 12,894.9	726.04	0.88/0.86
3	3/1/3	− 12,852.46	82.86	0.83/0.72/0.75
4	1/3/1/2	− 12,820.8	63.32	0.71/0.61/0.75/0.60
5	1/3/1/2/1	− 12,814.77	12.06	0.64/0.58/0.54/0.63/0.72
6	1/3/2/1/1/2	− 12,820.72	− 11.9	0.55/0.56/0.57/0.50/0.73/0.51
**Girls**				
1	3	− 10,745.75		100
2	3/3	− 10,516.03	459.44	18.1/81.8
3	1/3/3	− 10,482.06	67.94	0.82/0.70/0.74
4	1/2/3/3	− 10,457.74	49.28	0.66/0.67/0.74/0.71
5	2/1/3/1/1	− 10,467.42	− 20.84	0.55/0.60/0.62/0.64/0.70
6	2/1/1/3/1/2	− 10,471.63	− 8.42	0.61/0.64/0.60/0.63/0.66/0.54

^a^The best polynomial in each class is presented.

^b^BIC = Bayesian information criterion, smaller is better.

^c^Comparision between groups; Interpretation of 2ΔBIC = estimate of 2loge (B_10_). Evidence against simpler model: 0–2 = not worth mentioning; 2–6 = Positive; 6–10 = Strong; > 10 = Very strong.

^d^Posterior probability values greater than 0.70 indicate that the trajectory includes subjects with similar patterns of change.

**Table 2:** Trajectory descriptions, median posterior probabilities and growth parameter among boys, CFPS.

Group	N	Group membership (%)	Median (IQR) posterior probability	Growth parameter (standard errors)			
				Intercept	Linear	Quadratic	Cubic
Class 1	810	42.24	0.83 (0.70–0.97)	− 0.002 (0.346)	0.165 (0.111)	− 0.030 (0.011)	0.001 (0.000)
Class 2	857	41.55	0.72 (0.61–0.83)	2.557 (0.113)	− 0.176 (0.010)	–	–
Class 3	246	16.20	0.75 (0.60–0.90)	− 2.788 (0.582)	1.155 (0.190)	− 0.096 (0.020)	0.002 (0.001)

Three trajectories identified through latent class trajectory modeling with the TRAJ procedure using the censored normal model. The polynomials in age of Class 1 included linear, quadratic and cubic; the polynomials in age of Class 2 included linear; and the polynomials in age of Class 3 included linear, quadratic and cubic.

**Table 3:** Trajectory descriptions, median posterior probabilities and growth parameter among girls, CFPS.

Group	N	Group membership (%)	Median(IQR) posterior probability	Growth parameter (standard errors)			
				Intercept	Linear	Quadratic	Cubic
Class 1	941	52.98	0.82 (0.71–0.96)	0.751 (0.073)	− 0.106 (0.001)	–	–
Class 2	325	24.64	0.70 (0.54–0.86)	2.363 (0.483)	0.374 (0.173)	− 0.091 (0.019)	0.004 (0.001)
Class 3	341	22.38	0.74 (0.59–0.89)	− 1.851 (0.468)	1.095 (0.159)	− 0.112 (0.017)	0.003 (0.001)

Three trajectories identified through latent class trajectory modeling with the TRAJ procedure using the censored normal model. The polynomials in age of Class 1 included linear; the polynomials in age of Class 2 included linear, quadratic and cubic; and the polynomials in age of Class 3 included linear, quadratic and cubic.

### Baseline characteristics across different BMI z-scores trajectories

Table 4 presents the baseline characteristics across three BMI z-scores trajectories among boys and girls. At baseline, there was a significant difference in birth weight and single child between different BMI z-scores trajectories among boys (*p* < 0.05). Breastfeeding duration, family income, mother’s education, living location, community context degree, and social integration were significantly different between different BMI z-scores trajectories among girls (*p* < 0.05).

**Table 4:** Baseline characteristics of the population-based sample and analysis sample, CFPS.

Variables	Boys (N = 1913)					Girls (N = 1607)				
	Class 1	Class 2	Class 3	*P*	Missing	Class 1	Class 2	Class 3	*P*	Missing
**Early individual factors**										
**Birth weight**				0.025	211				0.936	187
Macrosomia	80 (11.03)	112 (14.89)	43 (19.11)			59 (6.99)	19 (7.04)	26 (8.50)		
Normal	603 (83.17)	603 (80.19)	172 (76.44)			730 (86.49)	233 (86.30)	261 (85.29)		
Low birth weight	42 (5.79)	37 (4.92)	10 (4.44)			55 (6.52)	18 (6.67)	19 (6.21)		
**Preterm birth**				0.724	19				0.911	18
Yes	33 (4.12)	30 (3.53)	11 (4.51)			39 (4.18)	15 (4.72)	14 (4.15)		
No	768 (95.88)	819 (96.47)	233 (95.49)			895 (95.82)	303 (95.28)	323 (95.85)		
**Breastfeeding duration**				0.626	–				0.004	7
< 6 months	145 (17.90)	139 (16.22)	44 (17.89)			193 (20.57)	45 (13.89)	49 (14.50)		
≥ 6 months	665 (82.10)	718 (83.78)	202 (82.11)			745 (79.42)	279 (86.11)	289 (85.50)		
**Family factors**										
**Family income**				0.056	228				0.012	172
Q1 (Low)	341 (47.96)	382 (50.26)	92 (42.99)			397 (46.16)	166 (58.25)	141 (48.62)		
Q2	150 (21.10)	163 (21.45)	37 (17.29)			185 (21.51)	58 (20.35)	59 (20.34)		
Q3	122 (17.16)	133 (17.50)	48 (22.43)			166 (19.30)	38 (13.33)	49 (16.90)		
Q4 (High)	98 (13.78)	82 (10.79)	37 (17.29)			112 (13.02)	23 (8.07)	41 (14.14)		
**Mother’s education**				0.213	449				< 0.001	363
Primary and below	300 (47.62)	346 (53.81)	90 (47.12)			308 (41.29)	149 (63.40)	134 (50.95)		
Junior/Senior High school	280 (44.44)	253 (39.35)	85 (44.50)			369 (49.46)	80 (34.04)	110 (41.83)		
Junior College and above	50 (7.94)	44 (6.84)	16 (8.38)			69 (9.25)	6 (2.55)	19 (7.22)		
**Living with parents**				0.341	–				0.078	–
Yes	489 (60.37)	503 (58.69)	157 (63.82)			569 (60.47)	175 (53.85)	209 (61.29)		
No	321 (39.63)	354 (41.31)	89 (36.18)			372 (39.53)	150 (46.15)	132 (38.71)		
**Single child**				0.004	–				0.135	–
Yes	412 (50.86)	443 (51.69)	154 (62.60)			505 (53.67)	154 (47.38)	173 (50.73)		
No	398 (49.14)	414 (48.31)	92 (37.40)			436 (46.33)	171 (52.62)	168 (49.27)		
**Community fators**										
**Living location**				0.189	–				< 0.001	–
Urban	288 (35.39)	279 (32.56)	94 (38.21)			380 (40.38)	77 (23.69)	117(34.31)		
Rural	522 (64.61)	578 (67.44)	152 (61.79)			561 (59.62)	248 (76.31)	224(65.69)		
**Community context degree**				0.744	20				< 0.001	21
Low	190 (23.66)	201 (23.76)	61 (25.00)			249 (26.80)	59 (18.32)	70 (20.90)		
Medium	385 (47.95)	384 (45.39)	116 (47.54)			424 (45.64)	129 (40.06)	166 (49.55)		
High	228 (28.39)	261 (30.85)	67 (27.46)			256 (27.56)	134 (21.61)	99 (29.55)		
**Social integration**				0.209	10				0.048	2
Actively	281 (34.91)	322 (37.79)	88 (35.77)			344 (6.63)	109 (33.54)	109 (31.96)		
Rarely	364 (45.22)	336 (39.44)	105 (42.68)			408 (43.45)	130 (40.00)	164 (48.09)		
None	160 (19.88)	194 (22.77)	53 (21.54)			187 (19.91)	86 (26.46)	68 (19.94)		

Number and percentage (N, %) are presented.

### Association between BMI z-scores trajectories and early individual, family and community factors among boys

Table 5 summarizes the results from the multivariate logistic regression examining characteristics associated with trajectories membership among boys. Controlling for multivariate, boys living with parents (OR 1.5; 95% CI 1.080–2.082), macrosomia (OR 1.772; 95% CI 1.188–2.644) and being a single child (OR 2.038; 95% CI 1.453–2.859) were risk factors for belonging in Class 3 trajectory than Class 1 trajectory. Boys with none social integration (OR 1.32; 95% CI 1.018–1.711) were more likely to belong in Class 2 trajectory than Class 1 trajectory.

**Table 5:** Odds Ratios and 95% Confidence Intervals for early individual, family and community factors related to BMI z-scores trajectories membership among boys, CFPS.

Variables	Class 2			Class 3		
	Model 1^a^	Model 2^b^	Model 3^c^	Model 1	Model 2	Model 3
**Early individual fators**						
**Birth weight**						
Macrosomia	1.309 (0.984–1.743)	1.315 (0.984–1.756)	1.312 (0.981–1.753)	1.317 (0.794–2.184)	1.624 (1.097–2.403)*	1.772 (1.188–2.644)*
Normal	1	1	1	1	1	1
Low birth weight	0.924 (0.584–1.46)	0.894 (0.563–1.42)	0.899 (0.565–1.431)	0.806 (0.326–1.991)	0.78 (0.379–1.606)	0.864 (0.415–1.802)
**Preterm birth**						
No	1	1	1	1	1	1
Yes	0.908 (0.532–1.551)	0.95 (0.554–1.628)	0.955 (0.556–1.639)	1.245 (0.591–2.623)	1.259 (0.592–2.679)	1.262 (0.592–2.687)
**Breastfeeding duration**						
< 6 months	1	1	1	1	1	1
≥ 6 months	1.113 (0.861–1.439)	1.095 (0.843–1.422)	1.120 (0.860–1.458)	0.988 (0.680–1.437)	1.040 (0.710–1.524)	1.052 (0.715–1.547)
**Family factors**						
**Family income**						
Q1 (Low)		1	1		1	1
Q2		1.114 (0.847–1.467)	1.119 (0.849–1.475)		0.828 (0.534–1.284)	0.825 (0.532–1.281)
Q3		0.976 (0.738–1.29)	0.985 (0.743–1.306)		1.137 (0.752–1.721)	1.133 (0.746–1.72)
Q4 (High)		0.867 (0.645–1.164)	0.868 (0.643–1.171)		1.154 (0.749–1.777)	1.144 (0.737–1.776)
**Mother’s education**						
Primary and below		1	1		1	1
Junior/Senior High school		0.794 (0.638–0.986)*	0.806 (0.644–1.009)		0.778 (0.562–1.078)	0.777 (0.556–1.087)
Junior College and above		0.856 (0.574–1.277)	0.857 (0.565–1.3)		0.614 (0.341–1.107)	0.608 (0.33–1.122)
**Living with parents**						
No		1	1		1	1
Yes		0.916 (0.739–1.134)	0.933 (0.750–1.161)		1.497 (1.088–2.060)*	1.500 (1.080–2.082)*
**Single child**						
No		1	1		1	1
Yes		1.145 (0.915–1.433)	1.146 (0.914–1.436)		2.041 (1.458–2.855)*	2.038 (1.453–2.859)*
**Community fators**						
**Living location**						
Urban			1			1
Rural			1.066 (0.847–1.342)			0.998 (0.711–1.401)
**Community context degree**						
Low			1			1
Medium			0.904 (0.702–1.166)			0.998 (0.688–1.449)
High			0.966 (0.724–1.288)			0.966 (0.626–1.49)
**Social integration**						
Actively			1.228 (0.986–1.529)			1.112 (0.801–1.542)
Rarely			1			1
None			1.32 (1.018–1.711)*			1.142 (0.776–1.681)

Class 1 was used as the reference group.

^a^Model 1: adjusted for early individual factors: preterm birth, birth weight, and breastfeeding duration.

^b^Model 2: adjusted for family factors: family income, mother education, living with parents and single child based on Model 1.

^c^Model 3: adjusted for community factors: living location, community context degree and social integration based on Model 2. *P < 0.05.

### Association between BMI z-scores trajectories and early individual, family and community factors among girls

Table 6 summarizes the results from the multivariate logistic regression examining characteristics associated with trajectories membership among girls. Girls’ mother with junior/senior high school education (OR 0.658; 95% CI 0.489–0.886) and living with parents (OR 0.704, 95% CI 0.524–0.946) were less likely to belong in Class 2 trajectory than Class 1 trajectory. In contrast, girls living in the advantaged communities (OR 1.539; 95% CI 1.052–2.252), rural-living (OR 1.558; 95% CI 1.133–2.142) and with none social integration (OR 1.496; 95% CI 1.07–2.091) were more likely to belong in Class 2 trajectory than Class 1 trajectory. Girls having a longer breastfeeding duration(≥ 6 months) (OR 1.469; 95% CI 1.035–2.087) were more likely to belong in Class 3 trajectory than Class 1 trajectory.

**Table 6:** Odds Ratios and 95% Confidence Intervals for early individual, family and community factors related to BMI z-scores trajectories membership among girls, CFPS.

Variables	Class 2			Class 3		
	Model 1^a^	Model 2^b^	Model 3	Model 1	Model 2	Model 3
**Early individual fators**						
**Birth weight**						
Macrosomia	0.989 (0.603–1.621)	0.94 (0.57–1.552)	0.96 (0.58–1.59)	1.204 (0.762–1.901)	1.199 (0.757–1.899)	1.223 (0.771–1.94)
Normal	1	1	1	1	1	1
Low birth weight	0.987 (0.581–1.676)	0.881 (0.513–1.511)	0.81 (0.469–1.398)	0.957 (0.564–1.624)	0.931 (0.547–1.585)	0.902 (0.529–1.539)
**Preterm birth**						
No	1	1	1	1	1	1
Yes	1.144 (0.61–2.147)	1.228 (0.649–2.324)	1.259 (0.661–2.398)	1.11 (0.593–2.08)	1.142 (0.608–2.143)	1.166 (0.62–2.194)
**Breastfeeding duration**						
< 6 months	1	1	1	1	1	1
≥ 6 months	1.608 (1.130–2.287)*	1.401 (0.974–2.014)	1.349 (0.935–1.948)	1.536 (1.091–2.161)*	1.479 (1.043–2.097)*	1.469 (1.035–2.087)*
**Family factors**						1
**Family income**						
Q1 (Low)		1	1		1	1
Q2		0.738 (0.52–1.045)	0.796 (0.559–1.134)		0.683 (0.478–0.976)*	0.709 (0.495–1.015)
Q3		0.718 (0.498–1.036)	0.828 (0.569–1.205)		0.684 (0.472–0.992)*	0.72 (0.493–1.05)
Q4 (High)		0.586 (0.395–0.867)*	0.731 (0.485–1.102)		0.91 (0.63–1.314)	1.006 (0.686–1.475)
**Mother’s education**						
Primary and below		1	1		1	1
Junior/Senior High school		0.592 (0.444–0.791)*	0.658 (0.489–0.886)*		0.874 (0.658–1.162)	0.929 (0.694–1.243)
Junior College and above		0.514 (0.288–0.919)*	0.619 (0.341–1.122)		0.742 (0.44–1.252)	0.798 (0.468–1.359)
**Living with parents**						
No		1	1		1	1
Yes		0.633 (0.476–0.843)*	0.704 (0.524–0.946)*		1.039 (0.785–1.375)	1.059 (0.795–1.411)
**Single child**						
No		1	1		1	1
Yes		0.887 (0.658–1.195)	0.917 (0.676–1.245)		0.976 (0.731–1.304)	0.98 (0.731–1.314)
**Community factors**						
**Living location**						
Urban			1			1
Rural			1.558 (1.133–2.142)*			1.174 (0.878–1.572)
**Community context degree**						
Low			1			1
Medium			1.06 (0.739–1.52)			1.278 (0.92–1.776)
High			1.539 (1.052–2.252)*			1.183 (0.813–1.721)
**Social integration**						
Actively			1.044 (0.772–1.413)			0.804 (0.603–1.071)
Rarely			1			1
None			1.496 (1.07–2.091)*			0.973 (0.694–1.363)

Class 1 was used as the reference group.

^a^Model 1: adjusted for early individual factors: preterm birth, birth weight, and breastfeeding duration.

^b^Model 2: adjusted for family factors: family income, mother education, living with parents and single child based on Model 1.

^c^Model 3: adjusted for community factors: living location, community context degree and social integration based on Model 2. *P < 0.05.

## Discussion

This study was undertaken to identify BMI z-scores trajectories among Chinese children aged 2–18 in the recent birth cohort and to assess the association between BMI z-scores trajectories and potential determinants based on the social-ecological model. Four main findings are worthy of further attention and discussion.

We identified three distinct BMI z-scores trajectories among boys and girls from CFPS: Not-overweight (Class 1), Persistent rapid descending but overweight during pre-school age (Class 2), and Rapid rising up to school age and then become-overweight (Class 3). The three growth pattern classes based on the International Obesity Task Force (IOTF) standards show potential deviations from “ideal” weight values of children across age. In our study, 40–50% of boys and girls are consistently not overweight, and the proportion is smaller than other studies^26–28^, because we found a larger group (41.55% for boys, 24.64% for girls) with persistent rapid descending that means decreased-risk of overweight, suggesting that a high-risk overweight status may be changed in adolescence^6^. Also, we found that children with BMI z-scores having rapid rising during the pre-school age could have a stable pattern with overweight during puberty, especially among boys. Another study also found that the first 4 years of life is a critical developmental window where long-term growth patterns^26^. Hence, BMI z-scores trends need to be monitored during the pre-school age and pay attention to those at higher risk of later overweight obesity.

Besides, we found that early individual, family, and community factors were associated with different BMI z-scores trajectories. At the individual level, BMI z-scores trajectories are associated with the birth weight and the breastfeeding duration. Macrosomia is more likely to be overweight during puberty among boys. Previous studies have found that high birth weight was associated with an elevated risk of later obesity in adolescence, and this association was significant only among boys^29,30^. One possible reason may be the sexual difference in growth of body composition, bone and muscle growth during the prenatal period^31^. Girls with longer breastfeeding duration (≥ 6 months) have a higher risk of belonging to be overweight during puberty in this study. Another nationwide study in China also found that if children receive breastfeeding duration for too long, their BMI tends to be higher^32^. However, several previous studies of Western children have suggested that breastfeeding over six months may be associated with a lower BMI in children^33,34^. Further studies are needed to explore the relationship of breastfeeding with children’s BMI in China.

The family factors associated with BMI z-scores trajectories are mother’s education, single child, and living with parents. Boys as only-children have a high risk of become-overweight during puberty, consistent with the previous studies^35–37^. Only-children spend more time on sedentary activities, eating more frequently away from home, more western fast food, and drinking more soft drinks than children with siblings, making them more likely to become obese^37–39^. Girls with better educated mothers are less likely to be overweight during pre-school age, which may be related to reasonable parenting methods and awareness of their child’s weight status^40^. Living with parents is associated with a lower incidence of overweight/obesity during school-age among girls. Parents’ behavior is a critical source of modeling in dietary intake behavior for adolescent girls^41^, and parental engagement between 10 and 18 years was associated with a decline in obesity risk across adolescence^42^. However, boys living with parents have a higher risk for overweight/obesity during school-age, which may be related to the preference for sons over daughters in China^43–45^.

For community factors, community context degree and social integration have an influence on BMI z-scores trajectories. Girls’ BMI z-scores trajectories are affected by their community environment, which is in agreement with the results of other studies^20,33,46–48^. Boys are more likely to spend time with peers in another community or independently explore other areas, while girls may be restricted by their community, associated with less physical activity^33,20,35,22,46,48^. Girls living in most advantaged communities are more likely to belong to the persistent rapid descending but overweight during pre-school age trajectory. Some studies found that children’s residential environments are related to their early growth health^49^, and neighborhood socioeconomic status on obesity may be most salient before pre-adolescence^50^. We also found that boys and girls with none social integration are more likely to be overweight during pre-school age. Higher social integration has been found to be associated with spending fewer hours watching television, playing computer games, and engaging in social media^51^, which could reduce the risk of being overweight. In addition, rural-living girls are more likely to have higher BMI z-scores before school age. Along with rapid social, economic, and nutritional transitions in rural China^8^, more food has been accessible, and caloric intake has been greater than ever before^39^. Lack of professional health information and healthy lifestyle^52^, pre-school age children in rural are at greater risk of obesity and need more attention.

This study has several limitations. First, determining the optimal number of latent trajectories based group-based trajectory modeling, is a process guided by statistical fit indices and investigator’s discretion that might overestimate or underestimate the real number of trajectories^53^. Although this method has limitations, we try to select the optimal classification of trajectories according to optimal fitting evaluation criteria. Second, our sample was selected from a nationally representative data set but not every sample has observations of all ages, so there will be some missing values. The group-based trajectory modeling can make maximum use of all observations for fitting. Third, using self-reported weight and height to estimate BMI z-scores could have some outliers and bias the trajectories. We identified outliers according to WHO growth standards^54^ and regarded them as missing values, ensuring the reliability of data to the maximum extent. Small changes in the bias related to changing the reporter are unlikely to have a large effect on the overall polynomial function of each trajectory^55^. Fourth, the measure of community factors was based on the feelings and impressions about the members and communities, leading to possible errors or biases. We will use more objective indicators in future research. In addition, we could not adjust for all possible confounders concerning BMI z-scores, and our study did not distinguish between exclusive and partial breastfeeding^56^. Therefore, it remains possible that unrecognized confounders or those that are underestimated could be contributing to our results.

Despite these limitations, our study has the advantage of prospectively covering the whole period from childhood to adolescence. We identified three BMI z-scores trajectories among children aged 2–18 in the recently birth cohort. Moreover, under the conceptual framework of social-ecological theory, we found that early individual, family, and community factors are associated with BMI z-scores trajectories, and targeted interventions at individual, family, and community levels are vital.

## Conclusions

There are heterogeneous BMI z-scores trajectories of children aged 2–18, and pre-school age is a critical window that could predict the long-term growth patterns. BMI z-scores trends need to be monitored during pre-school age with a focus on those at higher risk of later overweight obesity. In addition, early individual factors, family, and community factors were associated with different BMI z-scores trajectories, and there are sex differences. Maternal health should be managed to control macrosomia risks, reducing the risk of overweight during school age in boys. Also, more attention should be paid to BMI z-scores of girls in rural and advantaged communities, and propose targeted measures to reduce the risk of being overweight pre-school age. Targeted interventions at individual, family, and community levels are vital.

## Methods

### Materials

This study uses data from five waves of the China Family Panel Studies (CFPS), which collect individual-, family-, and community-level longitudinal data. The baseline survey was conducted in 2010, using multistage probability proportional to size sampling. Counties/administrative equivalents were drawn from 25 selected provinces, and then communities were drawn from selected counties/administrative equivalents. The socioeconomic level was used as an indicator of implicit stratification at these two stages. At the third stage, 25 households were random drawn from each sampled community based on the onsite sampling frame, and members of every household were asked to participate in the survey. The baseline sample represents 95% of the Chinese population, for an approximately response rate of 79%^57^. The baseline sample in 2010 was then followed up in 2012, 2014, 2016, and 2018. Details of the sample design have been described in other study^58^ and the website http://www.isss.pku.edu.cn/cfps/index.htm?CSRFT=XO4E-OIM2-ATE7-TX17-UW8H-99K1-6C5C-6Y5E.

We focused on children and adolescents aged 2–18 years old from five waves of the CFPS (2010, 2012, 2014, 2016, and 2018). Children aged 2–10 years old (n = 4412) were included at the baseline, and followed in 2012, 2014, 2016 and 2018. We took outliers of the dependent variable based on World Health Organization (WHO) growth standards^54^ as missing values. Children with more than three observations of the dependent variable were kept, resulting in an analytic sample of 3520. Then, we linked the children’s survey variables to family and community variables.

### BMI z-scores trajectory

BMI z-scores trajectory is the dependent variable in this study, which often represents the physical growth and nutritional status of children and adolescents. Measured heights and weights for each child were obtained through self-reported and used to calculate BMI z-scores according to the IOTF standards^59^.

### Early individual factors

Early individual factors included preterm birth, birth weight, and breastfeeding duration. Preterm birth was classified into two categories through the question “What is the gestational age of the child?”. The answer was coded “Yes” when less than 9 months and the other was code “No”. Birthweight was categorized as “macrosomia (birthweight ≥ 4 kg)”, “normal (2.5 kg < birthweight < 4 kg)” and “low birth weight (birthweight < 2.5 kg)” through the question “How many kilograms does the child weigh at birth?”. Breastfeeding duration was a categorical variable with two groups: < 6 months; and ≥ 6 months.

### Family factors

Family factors included family income per year, mother’s education, living with parents and being a single child. Family income was divided into four quartiles (Q1–Q4), with Q1 and Q4 indicating the lowest and the highest incomes, respectively. Mother’s education included primary and below, junior/senior high school, and junior college and above. The single child was treated as a dichotomous variable (“Yes” and “No”). Living with parents was treated as a dichotomous variable (“Yes” and “No”).

### Community factors

Community factors included living location, community context degree and social integration. Living location included urban and rural. Community context degree was a composite measure generated of six questions involving economic conditions, cleanliness of the road, the members' spiritual outlook, the homogeneity of the members, housing density, and the architectural pattern. Each question is based on the investigators’ feelings and impressions about the members and communities to evaluate, with scores from 1 to 7 scores. Community context degree was categorized into three classes (low, medium, and high) using latent profile analysis^60^. Social integration was classified into three categories (actively, rarely, and none), through the question “How many times did your family interact with your neighbors in the last month?”.

### Statistical analysis

Two stages analysis approach was carried out to explore BMI z-scores trajectories of children in the recent birth cohort and the influencing factors of different trajectories. Firstly, group-based trajectory modeling with the censored normal model was used to identify BMI z-scores trajectories of children aged 2–18. Group-based trajectory modeling can identify distinctive clusters of individual trajectories within the population using maximum likelihood latent-class models in SAS PROC TRAJ^61^. Considering the sexual difference in the growth of boys and girls, we identified BMI z-scores trajectories for boys and girls, respectively. For each gender subgroup, latent class trajectory models from 1 to 6 classes were tested, allowing for a variety of different order polynomials in age (e.g., cubic, quadratic, linear) to determine the best fitting polynomial form. The optimal number of groups and shapes of trajectories were selected for the best fit to the data, as assessed by Bayesian Information Criterion (BIC), the change in BIC between models (estimate of logged Bayes factor-2ΔBIC) and the average posterior probabilities^62^. Usually, a better BIC is a smaller one and a tenfold difference in Bayes factor is considered meaningful. In addition, the average posterior probabilities ≥ 0.7 as a criterion for model selection. After reviewing model statistics, we chose the best and the most concise trajectory model, and the shape of each group’s trajectory and the probabilities of being in each group were output.

Secondly, multivariate logistic regression models were used to estimate odds ratios (OR) and 95% confidence intervals (CI) for early individual factors, family, community factors related to BMI z-scores trajectories membership. According to the social-ecological theory, we started with individual factors and gradually added family and community factors. Therefore, three statistical models were tested: in Model 1, we controlled only for early individual factors: preterm birth, birth weight and breastfeeding duration. In Model 2, we added family factors: family income, mother education, living with parents and single child based on Model 1. In Model 3, we added community factors: living location, community context degree and social integration based on Model 2. Multiple imputation was used to account for missing data in the independent variables^63^. We conducted the same multivariate logistic regression models for boys and girls, respectively. All statistical procedures were performed by using the SAS 9.4.

## Data Availability

The original databases of this study are available from the online site: http://www.isss.pku.edu.cn/cfps/index.htm?CSRFT=XO4E-OIM2-ATE7-TX17-UW8H-99K1-6C5C-6Y5E.
